# Costimulatory CD226 Signaling Regulates Proliferation of Memory-like NK Cells in Healthy Individuals with Latent *Mycobacterium tuberculosis* Infection

**DOI:** 10.3390/ijms232112838

**Published:** 2022-10-25

**Authors:** Oscar Murillo, Josimar Dornelas Moreira, Weshely Kujur, Karen Velasco-Alzate, Sumit Sen Santara, Nagarjun V. Konduru, Sachin Mulik

**Affiliations:** 1 Department of Pulmonary Immunology, Center for Biomedical Research, The University of Texas Health Science Center at Tyler, Tyler, TX 75708, USA; 2 Department of Cellular and Molecular Biology, Center for Biomedical Research, The University of Texas Health Science Center at Tyler, Tyler, TX 75708, USA; 3 Infectious Diseases and Immunology Division, CSIR-Indian Institute of Chemical Biology, Kolkata 700032, West Bengal, India

**Keywords:** *Mycobacterium tuberculosis*, latent TB infection, memory-like NK cells, CD226, cMyc, FOXO1, glycolysis

## Abstract

It is now widely accepted that NK cells can acquire memory, and this makes them more effective to protect against some pathogens. Prior reports indicate memory-like NK cells (mlNKs) in murine model of Mycobacterium tuberculosis (Mtb) as well as in healthy individuals with latent TB infection (LTBI). The increased expression of CD226 was evident in mlNKs from LTBI+ people after stimulation with γ-irradiated Mtb (γ-Mtb). We thus evaluated the contribution of costimulatory CD226 signaling in the functionality of mlNKs in LTBI+ people. We found that blockade of CD226 signaling using the antibody- or CRISPR/Cas9-mediated deletion of the CD226 gene in NK cells diminished the proliferation of mlNKs from LTBI+ people. Blocking CD226 signaling also reduced the phosphorylation of FOXO1 and cMyc expression. Additionally, cMyc inhibition using a chemical inhibitor reduced proliferation by mlNKs from LTBI+ people. Moreover, blocking CD226 signaling reduced glycolysis in NK cells, and the inhibition of glycolysis led to reduced effector function of mlNKs from LTBI+ people. Overall, our results provide a role for CD226 signaling in mlNK responses to Mtb.

## 1. Introduction

Natural killer (NK) cells contribute to antimicrobial defense and antitumor immunity [1,2,3,4,5]. NK cells perform this function via the secretion of cytokines such as IFN-γ, TNF-α or by the perforin-granzyme-mediated lysis of microbial infected cells and tumor cells. Initially recognized as part of a stereotyped innate immune mechanism, several reports have clearly shown that NK cells can acquire memory characteristics [6,7,8,9]. A role of memory-like NK cells (mlNKs) has been shown for cytomegalovirus [7,10], Zika virus [11,12], simian-human immunodeficiency virus (SHIV) [13], HIV [14], flu virus [15,16] as well as tuberculosis (TB) [17,18]. NK memory has also been reported against haptens [6] as well as a combination of IL12, IL15 and IL18 cytokines [8,9]. NK cell immunotherapy involving mlNKs is currently tested in various human cancers [19,20,21,22,23]. A deeper understanding of molecules and signaling pathways that result in the adoption of the memory program in NK cells could lead to therapeutic maneuvers to optimize the functional response of mlNKs.

NK cell activity is regulated by signals received through activating and inhibitory receptors [24,25]. The signals received through activating receptors such as NKG2D, NKG2C and NKP30 leads to NK cell activation [24,25]. Conversely, signals received through inhibitory receptors such as NKG2A and KLRG1 leads to NK cell inhibition [24,25]. Microbial infected cells may downregulate MHC and upregulate NK cell activating ligands such as MICA, MICB that triggers the NKG2D-mediated activation of NK cells [26]. NK cells become further activated by innate cytokines [21,27] including IL12, IL-15, IL-18, IL21 [17,28,29], type I interferons [30] and costimulatory signaling such as DNAM-1 [31,32,33]. This results in potent anti-pathogen or anti-tumor defense.

CD226 (also known as DNAM-1) signaling contributes to NK cell function including the antitumor activities of NK cells [32,33]. CD226 receptor binding to ligands including moieties from different viruses induces the phosphorylation of Vav1, PLCγ1 and PI3K leading to the activation of ERK and AKT kinases [33]. FOXO1, a negative regulator of NK cell function [34], is directly phosphorylated by AKT and/or SGK1. Recently, it was shown that CD226 signaling leads to the phosphorylation of FOXO1 and the cytoplasmic translocation of phosphorylated FOXO1 and its proteosomal degradation resulting in NK cell activation [33]. An absence of CD226 in Ly49H+ NK cells leads to the suppression of expansion capacity and memory generation after infection with mouse Cytomegalovirus (MCMV) [35]. mlNKs that formed after Zika virus infection displayed higher gene expression of *CD226* [11]. Prior reports indicated that CD27+CD56+ NK cells from LTBI+ people proliferated and produced more IFN-γ and this subset constitutes a memory-like NK cell population (mlNKs) [17]. We found the increased expression of CD226 in TB reactive mlNKs. Here, using peripheral blood mononuclear cells (PBMC) from LTBI+ people we investigated the role of costimulatory CD226 signaling in the expansion capacity as well as a function of mlNKs response to Mtb stimulation.

## 2. Results

### 2.1. Blockade of CD226 Signaling Reduces Proliferation by mlNKs from LTBI+ People

**Figure 1:**
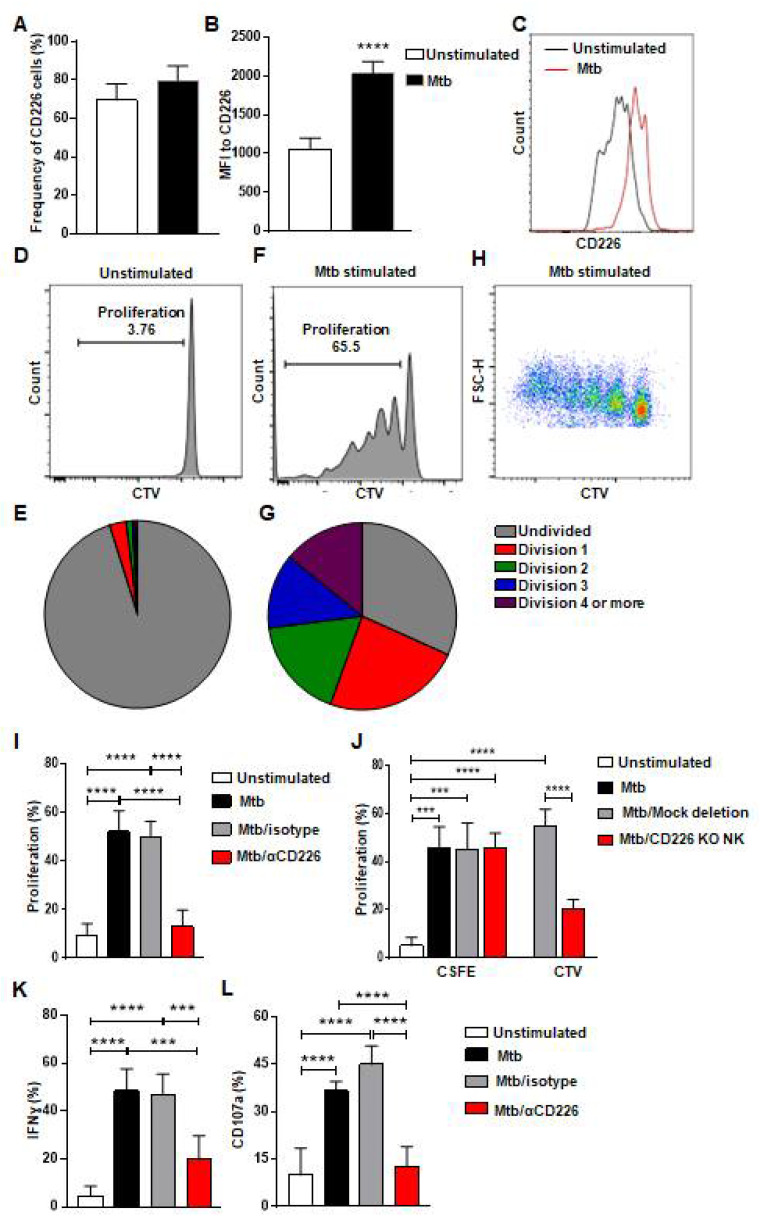
CD226 blockade reduces proliferation by mlNKs from LTBI+ people after stimulation with Mtb. (**A**) Frequency of CD226+ cells in CD3−CD45+CD56+CD27+ cells (n = 5). (**B**) MFI of CD226 in CD3−CD45+CD56+CD27+ cells (n = 5), (**C**) Comparative histogram. (**D**–**G**) Unstimulated CD3−CD45+CD56+CD27+ cells (**D**,**E**) CD3−CD45+CD56+CD27+ cells (**F**–**H**) were stimulated with γ-Mtb for 5 days. Pie charts show number of divisions in unstimulated (**E**) and stimulated cells (**G**). (**H**) Pseudo color plot showing the number of divisions in CD3−CD45+CD56+CD27+ cells stimulated with γ-irradiated Mtb. (**I**) Proliferation percentage in CD3−CD45+CD56+CD27+ cells unstimulated or stimulated with γ-Mtb or stimulated with treatments of control isotype or blocking CD226 antibody (n = 7). (**J**) Proliferation of CD3−CD45+CD56+ cells not deleted or deleted for *CD226* gene using CRISPR/Cas9 stained with cell trace violet (CTV) and co-cultured with autologous PBMC stained with CFSE and stimulated with γ-Mtb (n = 6). (**K**) IFN-γ, (**L**) CD107a positive cells in CD3−CD45+CD56+CD27+ cells unstimulated or stimulated with γ-Mtb or stimulated and treated with control isotype or blocking CD226 antibody (n = 5). Mean ± s.d. two-sided Student’s *t*-test or ANOVA. *** *p* < 0.001, **** *p* < 0.0001.

The co-stimulatory CD226 signaling contributes to NK cell function and tumor clearance [32,33]. Previous report indicates that CD226 signaling contributed to the expansion of Ly49H+ NK cells after MCMV infection and to the optimal differentiation of Ly49H+ memory NK cells [35]. It has been previously reported that CD226 expression was more on murine memory-like NK cells in a mouse model of TB [17] and these memory-like NK cells from LTBI+ donors proliferated and showed enhanced degranulation and IFN-γ production [17] (Figure S1A–D). We hypothesized that CD226 signaling contributes to the proliferation of mlNKs from LTBI+ people. To investigate this, we first stimulated peripheral blood mononuclear cells (PBMC) from LTBI+ (n = 5) people with γ-irradiated Mtb (γ-Mtb) for 5 days and assessed CD226 expression on mlNKs (Figure 1A–C). CD56+CD27+ mlNKs from LTBI+ donors (n = 5) proliferated in response to γ-Mtb stimulation (Figure 1D–H) compared to LTBI− (n = 6) people (Figure S1A–H) in accord with published report by others [17]. MFI of CD226 was higher on mlNKs after γ-Mtb stimulation, while frequencies of CD226+ mlNKs were comparable (Figure 1A–C). Next, we inhibited CD226 signaling using CD226 blocking antibody and the NK-cell-specific deletion of *CD226* gene using CRISPR/Cas9 (Figure S2A–C). Both approaches led to significantly reduced proliferation of mlNKs from LTBI+ people while isotype antibody had no measurable effect on NK cell proliferation (Figure 1I,J). Blocking CD226 antibody treatment also led to lesser IFN-γ production, degranulation as well as activation profile measured by CD69 and HLA-DR expression by mlNKs from LTBI+ people compared to isotype-antibody-treated counterparts (Figure 1K,L and Figure S1I–K). Blockade of CD226 with antibody also led to reductions in protein levels of IFN-γ, TNF-α, MIP-1β and MCP1 in the culture supernatants on day 5 when measured by ELISA (Figure S3A–E). Together, these results suggest a role of CD226 signaling in the proliferation of mlNKs from LTBI+ people (n = 5).

### 2.2. Inhibition of CD226 Signaling Leads to Reduction in FOXO1 Phosphorylation in NK Cells from LTBI+ People

**Figure 2:**
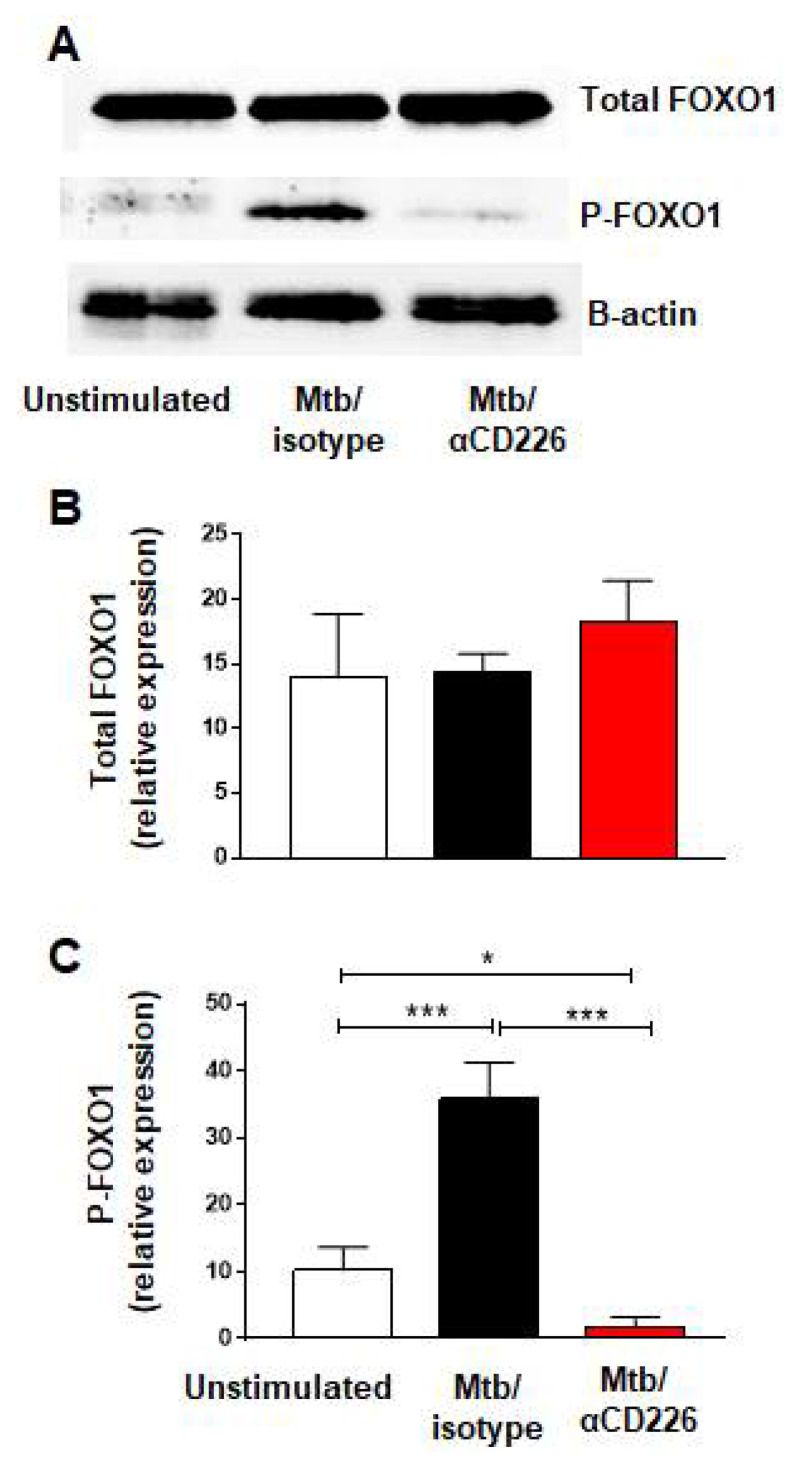
CD226 blockade reduced FOXO1 phosphorylation in NK cells from LTBI+ people after stimulation with Mtb. (**A**) Western blot from NK cells unstimulated or stimulated with γ-Mtb for 48 h and treated with isotype or blocking CD226 antibody. (**B**) Total FOXO1 and (**C**) phosphorylated FOXO1 (n = 5). Mean ± s.d. ANOVA. * *p* < 0.05, *** *p* < 0.001.

Recent report demonstrated that the activation of CD226 signaling led to the phosphorylation of FOXO1 and its proteasomal degradation [33]. FOXO1 is a negative regulator of NK cell function [34] thus CD226 signaling results in NK cell activation, by removing a negative regulator, FOXO1. We asked whether similar scenario occurs in NK cells from LTBI+ people when CD226 signaling is engaged. PBMC taken from LTBI+ people (n = 5) and stimulated with γ-Mtb were treated with blocking CD226 antibody or isotype matched antibody and NK cells were purified on day 2 to assess the phosphorylation of FOXO1. We noted the increased phosphorylation of FOXO1 in the isotype-antibody-treated group (Figure 2A–C). Conversely, blocking CD226 led to the lower phosphorylation of FOXO1, suggesting the increased presence of negative regulator FOXO1 in this group (Figure 2A–C). Overall, in agreement with a previous report in NK cells [33], these results indicate that blocking CD226 signaling reduced the phosphorylation of FOXO1 in NK cells from LTBI+ people (n = 5).

### 2.3. Inhibition of CD226 Signaling Reduces cMyc Levels in NK Cells from LTBI+ People

**Figure 3:**
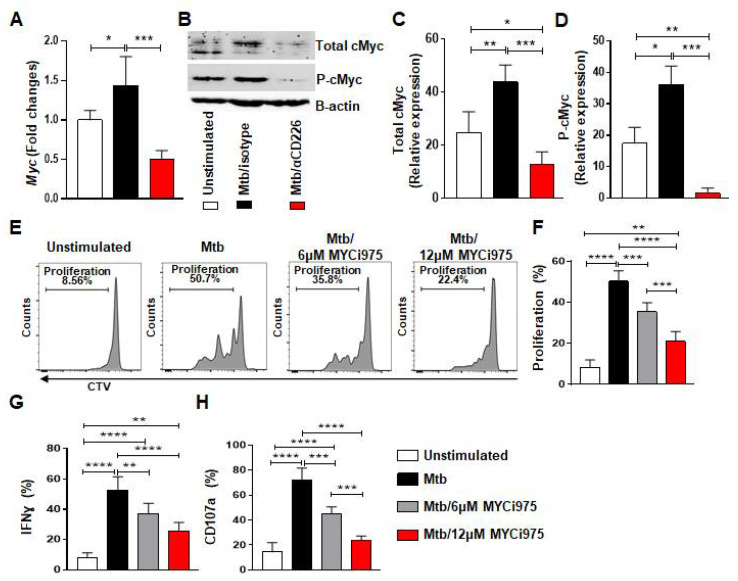
cMyc inhibition reduces effector function of mlNKs from LTBI+ people after stimulation with Mtb. (**A**) *Myc* gene expression by TaqMan PCR in NK cells after 24 h of stimulation with γ-Mtb and treatments with isotype or blocking CD226 antibody (n = 5). (**B**) Western blot for NK cells unstimulated or stimulated with γ-Mtb for 48 h and treated with isotype or blocking CD226 antibody (n = 5). (**C**) Total cMyc and (**D**) phosphorylated cMyc. (**E**–**H**) PBMC were stimulated with γ-Mtb or left unstimulated for 5 days. cMyc inhibitor CMYi975 (6 or 12 µM) or DMSO vehicle was added every other day after stimulation with γ-Mtb (**E**,**F**) Proliferation of mlNKs from LTBI+ donors (n = 5) stimulated with γ-Mtb and treated with CMYi975 (6 or 12 µM). (**G**) IFN-γ, (**H**) CD107a positive cells in CD3−CD45+CD56+CD27+ cells (n = 5) unstimulated or stimulated with γ-Mtb and treated with CMYi975 (6 or 12 µM). Mean ± s.d. ANOVA. * *p* < 0.05, ** *p* < 0.01, *** *p* < 0.001, **** *p* < 0.0001.

We next explored molecules involved in the proliferation of mlNKs that may be affected by CD226 blockade. cMyc is previously reported to be a target of XBP1s and integrates signals from endoplasmic reticulum (ER) stress to drive NK cell responses after MCMV infection and tumors [36]. PBMC from LTBI+ people (n = 5) were stimulated with γ-Mtb and treated with isotype antibody or CD226 blocking antibody. NK cells were purified at day 1 and day 2 post treatment. When we measured ER stress related genes (day 1 post treatment) such as *Ire1α*, *Xbp1*, we did not observe significant differences between isotype antibody and CD226 blocking antibody regimens (Figure S4B). While the expression levels of *Chop*, *Atf4* and *Bip* were elevated in blocking CD226 antibody groups (Figure S4B). We also measured molecules associated with Wnt signaling [37] and found no differences in *Tcf7* gene expression (Figure S4A). *Dll1* expression was elevated while *Jag1* expression was reduced upon CD226 blocking antibody groups (Figure S4A). *Axin 2* expression was reduced in both isotype and CD226 blocking antibody groups (Figure S4A). Notably, *Myc* gene which is associated with cellular proliferation was higher in isotype antibody group, while *Myc* expression was reduced when CD226 signaling was blocked (Figure 3A). We also validated these results using Western blotting (Figure 3B–D) performed on day 2 post treatment. Overall, these results indicate lower cMyc levels in NK cells after CD226 blockade.

### 2.4. Inhibition of cMyc Using a Chemical Inhibitor Reduces Proliferation by mlNKs from LTBI+ People

We investigated whether the inhibition of cMyc using a chemical inhibitor of cMyc (MYCi975) [38] impacts the proliferation of CD56+CD27+ mlNKs from LTBI+ people. To do this, PBMC from LTBI+ people (n = 5) were pretreated with cMyc inhibitor or mock for 30 min and stimulated with γ-irradiated Mtb. The inhibitor and mock were replenished on alternate days and the proliferation of CTV-labeled CD56+CD27+ mlNKs was assessed 5 days later by flow cytometry. The culture supernatants on day 5 were assessed for the quantification of cytokines (Figure S5A–E, n = 5). We observed a significant reduction in the proliferation and effector function of CD56+CD27+ mlNKs from LTBI+ people (n = 5) when cMyc was inhibited compared to mock-treated groups (Figure 3E–H and Figure S6A). cMyc inhibition also led to a reduction in the protein levels of IFN-γ, TNF-α, MIP-1β, MCP1 and IL-10 in the culture supernatants on day 5 when measured by ELISA (Figure S5A–E, n = 5). Overall, these results indicate a role for cMyc in the proliferation of CD56+CD27+ mlNKs from LTBI+ people in response to γ-Mtb stimulation.

### 2.5. Inhibition of CD226 Signaling Lowered Glycolysis in NK Cells, and Glycolysis Inhibition Reduced Proliferation by mlNKs from LTBI+ People

**Figure 4:**
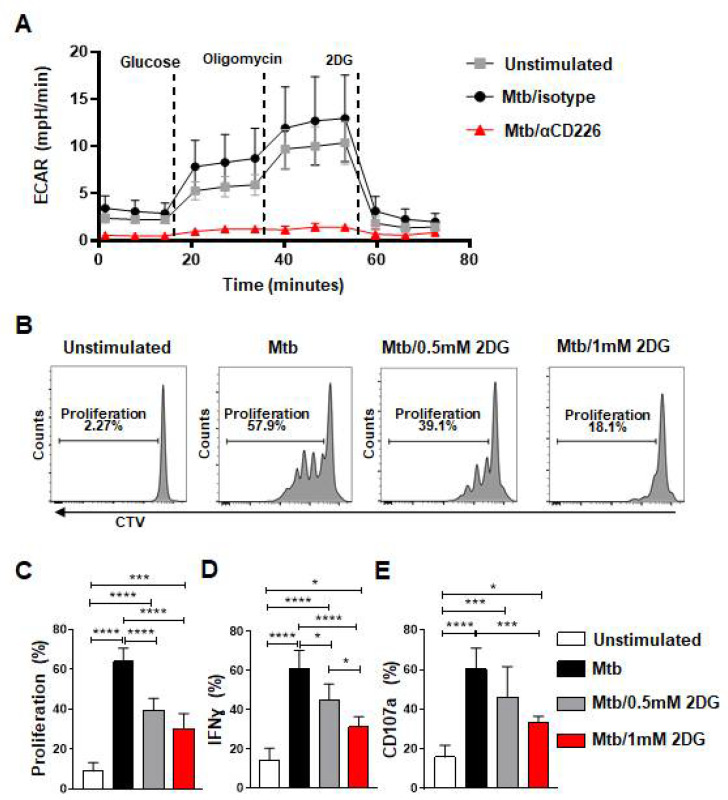
Blockade of CD226 reduced glycolysis in NK cells and glycolysis inhibition diminished effector function of mlNKs from LTBI+ donors after stimulation with Mtb. (**A**) PBMC from LTBI+ donors were stimulated with γ-Mtb for 48 h and treated with isotype or blocking CD226 antibody. After this, 5 × 10^5^ purified NK cells (purity > 90%) were plated to perform the glycolysis assay using seahorse analyzer (n = 4). (**B**) Chemical blockade of glycolysis using 2DG (0.5 or 1 mM every three days) after stimulation with γ-Mtb for 5-day time period was complete. (**B**–**E**) Proliferation, frequencies of IFN-γ+ cells and CD107a+ cells among CD3−CD45+CD56+CD27+ cells unstimulated or stimulated with γ-Mtb and treated with 2DG (n = 5). Mean ± s.d. ANOVA. * *p* < 0.05, *** *p* < 0.001, **** *p* < 0.0001.

cMyc has been shown to regulate glycolysis in various cells [39,40,41,42]. Since CD226 blockade reduced cMyc levels, we probed whether the inhibition of CD226 signaling modulates the glycolytic metabolism of NK cells from LTBI+ people. PBMC from LTBI+ donors (n = 4) were stimulated with γ-Mtb and treated with isotype antibody or CD226 blocking antibody. NK cells were purified at day 2 post treatment and subjected to measure glycolysis (extracellular acidification rate) by Seahorse analyzer. We found reduced glycolysis in NK cells from LTBI+ people when CD226 signaling was inhibited (Figure 4A and Figure S6C–E). Glycolysis inhibition has been shown previously to impact NK cell proliferation and IFN-γ production [43]. To determine if the inhibition of glycolysis impacts the function of CD56+CD27+ mlNKs from LTBI+ people, PBMC from LTBI+ people (n = 5) were stimulated with γ-Mtb and treated with mock or 2DG (glycolysis inhibitor) [43]. The proliferation and function of CD56+CD27+ mlNKs were assessed 5 days later. We observed a significant reduction in the proliferation of CD56+CD27+ mlNKs from LTBI+ people when glycolysis was inhibited compared to mock-treated groups (Figure 4B,C and Figure S6B). We also observed a reduction in IFN-γ production and degranulation by CD56+CD27+ mlNKs from LTBI+ people from 2DG-treated groups compared to mock treatment (Figure 4D,E). Together, these results indicate a role for glycolysis in the proliferation of CD56+CD27+ mlNKs from LTBI+ people in response to γ-Mtb stimulation.

## 3. Discussion

In this report, we show that co-stimulatory signaling through CD226 contributes to the function of mlNKs in LTBI+ people. Our results show that the blockade of CD226 signaling using a blocking antibody or CRISPR/Cas9-mediated *CD226* deletion reduced proliferation by CD56+CD27+ mlNKs from LTBI+ people in response to γ-Mtb stimulation. Furthermore, our results revealed that blocking CD226 signaling reduced the phosphorylation of FOXO1 and lowered cMyc levels. We also showed that the inhibition of cMyc using a chemical inhibitor reduced proliferation by CD56+CD27+ mlNKs from LTBI+ people in response to γ-Mtb stimulation. Finally, we show that CD226 blockade lowered glycolysis in NK cells and inhibiting glycolysis reduced proliferation by CD56+CD27+ mlNKs from LTBI+ people in response to γ-Mtb stimulation. Overall, our results suggest a role of costimulatory signaling through CD226 in the expansion capacity and function of CD56+CD27+ mlNKs from LTBI+ people and could imply that defects in CD226 signaling may influence mlNKs function in human and possibly Mtb infection outcomes.

Prior report showed a critical role of the ER stress sensor IRE1α and its substrate transcription factor XBP1 in promoting NK cell effector responses to fend off virus infection and to clear tumors [36]. These antiviral and anti-tumor effects were mediated by cMyc, a molecule implicated in cellular proliferation [36]. We noted in LTBI+ people that the expression of *Ire1α*, *Xbp1* did not vary between isotype and CD226 blocking antibody treatment regimens suggesting ER stress may not be involved in the regulation of mlNK responses in LTBI+ persons. Multiple reports have advocated a role for Wnt signaling in proliferation by memory-like NK cells [11,14]. Our results show the equivalent expression of multiple Wnt signaling genes (*Axin2*, *Tcf7*) in CD226-antibody-treated versus isotype-treated NK cells from LTBI+ people.

cMyc regulates proliferation in diverse cell types [36,37,44,45,46]. In line with these studies, we report reduced mlNKs proliferation with CD226 blockade due to diminished cMyc levels. Our finding of the inhibition of CD56+CD27+ mlNKs proliferation and effector function from LTBI+ people after treatment with cMyc inhibitor is also consistent with cMyc promoting proliferation and effector function in NK cells as also noted by others [36,47]. However, it is unclear how CD226 signaling promotes cMyc expression in mlNKs cells which we are currently investigating. One likely effect involves effects on aspect of immunometabolism. In fact, some reports do indicate a critical role of cMyc in modulating metabolic process [39,40,41]. Some show that cMyc promotes glycolysis [39,40,41], but others suggest its role in oxidative phosphorylation and mitochondrial metabolism [42]. It was not established if CD226 signaling modulates metabolism in NK cells. Recently, CD226 signaling was reported to promote glycolysis in endothelial cells [48]. Similarly, we noted diminished glycolysis in NK cells from LTBI+ people after CD226 blockade. It is possible that inhibiting CD226 signaling exerts its negative effect on glycolytic process via reduction in cMyc activity. In addition to NK cells, CD226 signaling can also regulate CD8 T cell function [49]. In fact, blocking PD-1 and inhibiting CD226 function compromise tumor clearance by CD8 T cells [50,51,52]. It may be worthwhile to see whether CD226 signaling regulates cMyc expression/glycolytic pathway in CD8 T cells that are actively clearing the tumors. We speculate that the outcome is likely to be positive. Overall, the results from our current NK cell study suggest that CD226 signaling may also play a role in regulating the metabolism of NK or even CD8 T cells in other contexts such as infection or tumor settings.

### Limitations

Due to limited numbers of CD56+CD27+mlNKs, our experiments involved probing the protein levels of cMyc, FOXO1 or glycolysis assays in total NK cells after stimulation of the PBMC from LTBI+ people with γ-Mtb and treatments with isotype or blocking CD226 antibodies. We noted reduced CD56+CD27+ mlNKs proliferation from LTBI+ people after stimulation with γ-Mtb and treatments with cMyc inhibitor or glycolysis inhibitor (2DG). Thus, we speculate that CD226 inhibition also reduces FOXO1, cMyc levels or glycolysis in CD56+CD27+ mlNKs from LTBI+ people. Due to human primary cell studies with in vitro nature, the findings from this study require further studies with murine models with *CD226* gene deletion where we believe that the absence of the CD226 gene may lead to multiple defects in NK and T cell compartment leading to increased susceptibility to Mtb.

## 4. Materials and Methods

### 4.1. Ethics Statement

All human samples studies were approved by the Institutional Review Board of the University of Texas Health Science Center at Tyler with protocol number 2021-033. For this study we collected blood samples from healthy volunteers for the isolation of PBMC.

### 4.2. Sample Collection

All donors were well-informed about the study before the acquisition of the informed consent. For the study blood from healthy persons positive to QuantiFERON-TB Gold tests and healthy persons negative to QuantiFERON-TB Gold tests were collected. All subjects were between 25 and 55 years old, including both genders.

### 4.3. Isolation and Culture of PBMC and CD226 Blockade

The blood was mixed with RPMI-1640 (Cytiva, Salt Lake City, UT, USA) complete media with 10% fetal bovine serum (FBS) (HyClone, Salt Lake City, UT, USA) and layered over Ficoll-Paque^TM^ plus gradient (Cytiva). After 25 min of centrifugation (with minimal acceleration, no brake, 1650 rpm at 25 °C), PBMC were collected and washed 2 times with RPMI-1640 (Cytiva, Salt Lake City, UT, USA) complete media with 10% FBS (HyClone, Salt Lake City, UT, USA). PBMC were cultured at 2.5 × 10^6^ cells/well in 24-well plates with RPMI-1640 (Cytiva, Salt Lake City, UT, USA) with 10% AB heat-inactivated human serum (Sigma-Aldrich, Burlington, MA, USA) at 37 °C and 5% CO_2_. PBMC were treated with blocking anti-human CD226 mAb (clone 11A8) at 10 µg/mL (BioLegend, San Diego, CA, USA) or isotype control antibody (10 µg/mL, BioLegend) one hour before stimulation with γ-Mtb H37Rv at 10 µg/mL. The control or blocking antibodies were supplemented every other day. After 48 h to 5 days of stimulation, flow cytometry, real time PCR and metabolism assays were performed.

### 4.4. NK Cell Labelling with Cell Trace Violet/CFSE

PBMC or NK cells were labelled with CellTrace™ Violet (CTV, Invitrogen, Waltham, MA, USA) 1 µM, or CFSE (eBioscience, San Diego, CA, USA) 1 µM for 20 min at 37 °C, followed by multiple washes, and cells were seeded at 2.5 × 10^6^ cells/well.

### 4.5. c-Myc and Glycolysis Inhibition

PBMC from LTBI+ donors were cultured in 24-well plates (2.5 × 10^6^ cells/well) with RPMI-1640 (Cytiva) complete media with 10% AB heat-inactivated human serum (Sigma-Aldrich, Burlington, MA, USA) at 37 °C and 5% CO_2_. The cells were treated with MYCi975 (6 µM, 12 µM or 18 µM, MedChemExpress, Monmouth Junction, NJ, USA) or 2-DeoxyGlucose (2DG, 0.5 mM and 1 mM, Sigma-Aldrich) or control DMSO for 30 min. After this time, the cells were stimulated with γ-Mtb (10 µg/mL) for 5 days and analyzed by flow cytometry. cMyc inhibitor was supplemented every other day while 2DG was supplemented every three days.

### 4.6. Flow Cytometry

PBMC were stained with PerCP anti-human CD45 (clone: HI30, BioLegend), APC-Cy7 anti-human CD3 (clone: HIT3a, BioLegend), BV605 anti-human CD56 (clone: HCD56, BioLegend), PE anti-human/mouse/rat CD27 (clone: LG.3A10, BioLegend), Alexa Fluor 647 anti-human CD226 (DNAM-1) (clone: DX11, BD Biosciences, San Diego, CA, USA) and Zombie Red™ Fixable Viability Kit (live/dead staining) (BioLegend) in 1X PBS at 4 °C for 20–30 min. The cells were washed three times and cell acquisition was performed on LSRFortessa X-20 (BD Biosciences).

For intracellular staining, surface staining was followed by cellular permeabilization with Fixation/Permeabilization solution according to the manufacturer’s protocol (BD biosciences). The cells were washed with Perm/Wash buffer and were stained for FITC anti-human IFN-γ (clone: 45.B3, BioLegend) and APC anti-human CD107a (clone H4A3, BioLegend). In these assays, Brefeldin A (BD Biosciences) was added 4 h prior to surface staining, and CD107a antibody was added 2 h before Brefeldin A. The cellular acquisition was performed on LSRFortessa X-20 and data analysis was conducted using FlowJo v10 software (TreeStar, Ashland, OR, USA).

### 4.7. Relative Quantification of Genes by Real Time PCR

RNA isolation was performed using RNeasy mini kit (Qiagen, Germantown, MD, USA) according to manufacturer’s protocol and cDNA synthesis was carried out using High-Capacity cDNA Reverse Transcription Kit (Applied Biosystems, Waltham, MA, USA). TaqMan probes (Thermo fisher, Waltham, MA, USA) for endoplasmic reticulum stress genes: E*rn1* (Hs00980095_m1), *Ddit3* (Hs01090850_m1), *Atf4* (Hs00909569_g1), *Hspa5* (Hs00607129_gH), *Xbp1* (Hs00231936_m1) *Xbp1s* (Hs03929085_g1), and proliferative genes: *Myc* (Mm00487804_m1), *Axin2* (Mm00443610_m1), *Dll1* (Mm01279269_m1), *Tcf7* (Mm00493445_m1), and *Jag1* (Mm00496902_m1) genes were used. To perform relative quantification, all samples were normalized with *Gapdh* (Mm99999915_g1) gene. The samples were amplified with TaqMan^®^ gene expression master mix (Applied biosystem) in the QuantStudio 7 flex machine (Applied biosystem).

### 4.8. Metabolism Assays Using Seahorse Analyzer

Human PBMC from LTBI+ donors were treated with blocking anti-human CD226 clone 11A8 (10 µg/mL, BioLegend) or isotype control antibody (10 µg/mL, BioLegend) for 1 h at 37 °C and 5% CO_2_. The cells were stimulated with γ-Mtb or left untreated for 48 h at 37 °C and 5% CO_2_. NK cells were enriched using human NK isolation kit (Miltenyi Biotec, Gaithersburg, MD, USA. purity more than 90%). NK cell glycolytic status or extracellular acidification rate (ECAR) was measured via extracellular flux assay using Seahorse XFe96 Analyzer (Agilent Technologies, Santa Clara, CA, USA). NK cells were washed and resuspended in Agilent XF assay medium supplemented with 1 mM L-Glutamate and then plated for the assay at a density of 5 × 10^5^ NK cells/well in a 96-well Seahorse plate coated with Poly-L-Lysine. During the assay glucose (10 mM), oligomycin (1 µM) and 2DG (50 mM) were injected according to the manufacturer’s protocol. Basal glycolysis, glycolytic capacity and glycolytic reserve were calculated using Seahorse Wave 2.6.3 Desktop Software, Agilent Technologies, USA.

### 4.9. CRISPR/Cas9-Mediated Deletion of CD226 Gene

NK cells isolated from PBMC were kept with human IL-15 (10 ng/mL) (BioLegend) for 24 h. After this, 2–5 × 10^6^ NK cells were resuspended in 90 microliters Nucleofector^TM^ solution and 10 microliters sgRNA-Cas9 complex was added (0.3 pmol of each sgRNA and 40 pmol spCas9). Four *CD226* sgRNAs (Synthego, Redwood City, CA, USA) designed from *CD226* human gene (CD226+69947022, CD226+69947028, CD226+69947029, and CD226-69947061) were used. spCas9 protein was also procured from Synthego. NK cells were electroporated using Amaxa^TM^ human NK cell nucleofector^TM^ kit (Lonza, Bend, OR, USA) according to the manufacturer’s protocol. After electroporation, NK cells were maintained with 10 ng/mL human IL-15 for 5 days, and the deletion of *CD226* was verified by Western blotting and flow cytometry. *CD226* deleted or control NK cells were stained with CTV and co-cultured with PMBC isolated from the same donor that were stained with CFSE. Proliferation was measured by flow cytometry after the stimulation of co-cultured cells with γ-irradiated Mtb.

### 4.10. Western Blotting

The protein expression and phosphorylation of c-Myc and FOXO1 were determined by Western blotting using whole cell protein extracts from 10^6^ NK cells lysed with RIPA buffer with protease and phosphatase inhibitors (G-Biosciences, St Louis, MO, USA). The samples were run in 10% SDS-PAGE gel and transferred onto nitrocellulose membranes, which were blocked with 5% skimmed milk in TBST for 1h. After this, the membranes were incubated overnight at 4 °C with primary antibodies for cMyc (Cell signaling, 1:1000 dilution), pcMyc (Cell signaling, 1:1000 dilution), FOXO1 (Cell signaling, 1:1000 dilution), pFOXO1 (Cell signaling, 1:1000 dilution), β-actin (Cat# 4967, Cell signaling, 1:1000 dilution). The membranes were incubated for 1h with secondary antibody conjugated with HRP and proteins bands were visualized by Clarity ECL substrate (Bio-Rad, Hercules, CA, USA) followed by capturing with ChemiDoc™ Imaging System (Bio-Rad).

### 4.11. Statistical Analysis

GraphPad Prism 7 software was used for experimental analysis. The unpaired or paired t-test or ANOVA was used for determining the statistical significance at * *p* ≤ 0.05, ** *p* ≤ 0.01, *** *p* ≤ 0.001 and **** *p* ≤ 0.0001.

## Data Availability

The manuscript contains all the data including the supplemental figures.

## Supplementary Materials

The following supporting information can be downloaded at: https://www.mdpi.com/article/10.3390/ijms232112838/s1.
