# Supplementary material for: Costimulatory CD226 Signaling Regulates Proliferation of Memory-like NK Cells in Healthy Individuals with Latent Mycobacterium tuberculosis Infection
Source: Int J Mol Sci. 2022 Oct 25;23(21):12838. doi: 10.3390/ijms232112838 (PMC9653912; doi:10.3390/ijms232112838)
Supplement: Supplementary file 1 [file ijms-23-12838-s001.zip › ijms-1946004-supplementary.pdf]

Figure S1. CD56+CD27+ cells from LTBI+ donors proliferate after stimulation with Mtb. A. Gating strategy used to analyze memory like NK cells. **B**. proliferation, **C**. frequencies of IFN-γ+ cells and **D**. frequencies of CD107a+ cells in CD3-CD45+CD56+CD27+ cells from PBMC of LTBI+ and LTBI- donors stimulated with γ-irradiated Mtb (n=6). **E-H**. Proliferative profile of non-memory CD56+CD27- cells or memory like CD56+CD27+ cells from LTBI- (**E,F**) or LTBI+ (**G,H**) after stimulation with γ-irradiated Mtb for 5 days. **I-K.** PBMC from LTBI+ people were stimulated with ɣ-Mtb and treated with isotype or blocking CD226 antibody. On day 5, frequencies and MFI for CD69, CD16 and HLA-DR in CD56+CD27+ NK cells were determined. n=4. of Mean ± s.d. two-sided Student's t-test. *p< 0.05, **p< 0.01, ***p< 0.001, ****p< 0.0001.

Figure S2. *CD226* gene deletion strategy and verification. A. Purified NK cells (CD3-CD45+CD56+ cells) were deleted or not for *CD226* gene using CRISPR/Cas9 technology (Synthego) and maintained for 5 days in recombinant human IL-15. **B,C** Validation of gene deletion was performed using flow cytometry (**B**) and western blotting (**C**).

Figure S3. Blocking CD226 antibody reduces expression of several cytokines in supernatant of PBMC stimulated with Mtb. PBMC from LTBI+ donors were stimulated with γ-irradiated Mtb for 5 days and treated with isotype antibody or CD226 blocking antibody every other day. The supernatant was analyzed for **A**. IFN-γ, **B**. TNFα, **C**. MIP-1β, **D**. MCP-1 and, **E**. IL-10 by ELISA to determine differential cytokine profile (n=5). Mean ± s.d. ANOVA. *p< 0.05, **p< 0.01, ***p< 0.001, ****p< 0.0001.

Figure S4. Gene expression of Wnt/TCF1 pathway and ER stress genes. Enriched NK cells after 24 hours of stimulation with γ-irradiated Mtb and treatments with isotype or CD226 blocking antibody were probed for quantification of genes using TaqMan probes. Expression of Wnt/TCF1 pathway genes (**A**) (*Tcf7*, *Dll1*, *Jag1*, *Axin2*) and ER stress genes (**B**) (*Chop*, *Atf4*, *BiP*, *IRE1α*, *Xbp1* and spliced *XBP1*) (n=5). Mean ± s.d. ANOVA. *p< 0.05, **p< 0.01.

Figure S5. cMyc inhibition with MYCi975 reduces expression of several cytokines in supernatant of PBMC stimulated with Mtb. PBMC from LTBI+ donors were stimulated for 5 days and treated with 6uM and 12uM of cMyc inhibitor MYCi975 every other day or DMSO vehicle. The supernatant was analyzed for **A**. IFN-γ, **B**. TNFα, **C**. MIP-1β, **D**. MCP-1 and, **E**. IL-10 by ELISA to determine differential cytokine profile (n=5). Mean ± s.d. ANOVA. *p< 0.05, ***p< 0.001, ****p< 0.0001.

Figure S6. NK cells viability after cMyc inhibitor MYCi975 and 2DG treatments and effect of CD226 blockade on glycolysis. A. PBMC from LTBI+ donors were stimulated for 5 days with γ-irradiated Mtb and treated with 6 uM, 12 uM and 18 uM of cMyc inhibitor MYCi975 or mock every other day, and viability of CD3-CD45+CD56+CD27+ cells was determined by flow cytometry (n=5). **B**. PBMC from LTBI+ donors were stimulated for 5 days with γ-irradiated Mtb and treated with 0.5 mM, 1 mM, 10 mM and 20 mM of glycolysis inhibitor 2DG or mock every three days, and viability of CD3-CD45+CD56+CD27+ cells was determined by flow cytometry (n=5). **C-E** PBMC from LTBI+ donors were stimulated with γ-irradiated Mtb for 48 hours and treated with isotype or blocking CD226 antibody. After this, $5x10^5$ enriched NK cells were plated to perform glycolysis assay using seahorse analyzer. **C**. Basal glycolysis, **D**. glycolytic capacity and **E**. glycolytic reserve were determined (n=4). Mean ± s.d. ANOVA. *p< 0.05, **p<0.01.

Figure S1

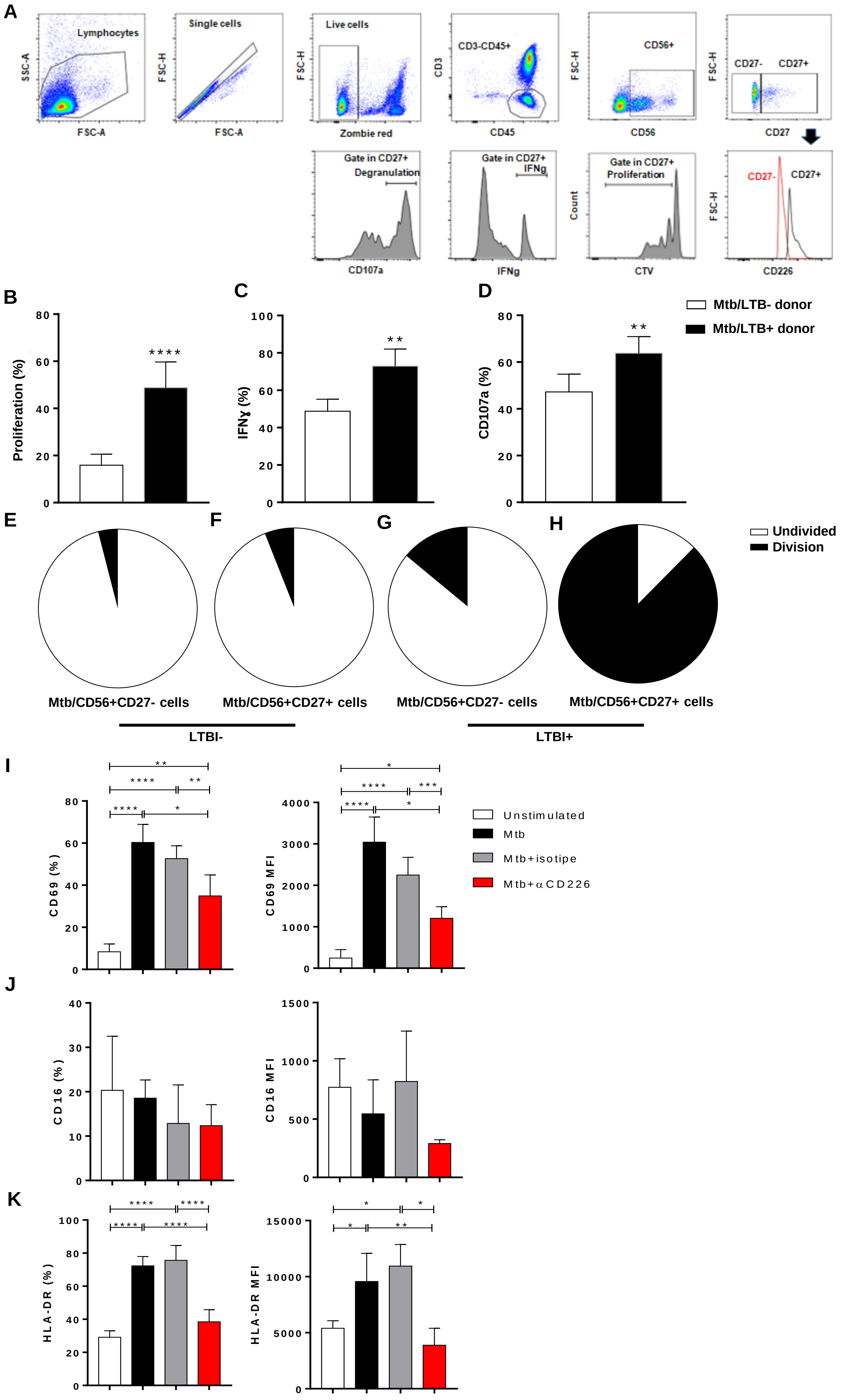
A
Lymphocytes
SSC-A
FSC-A
Single cells
FSC-H
FSC-A
Live cells
FSC-H
Zombie red
CD3-CD45+
CD3
CD45
CD56+
FSC-H
CD56
CD27-
CD27+
FSC-H
CD27
Gate in CD27+
Degranulation
CD107a
Gate in CD27+
IFNg
IFNg
Gate in CD27+
Proliferation
Count
CTV
CD27-
CD27+
FSC-H
CD226
B
Proliferation (%)
****
C
IFNɣ (%)
**
D
CD107a (%)
**
Mtb/LTB- donor
Mtb/LTB+ donor
E
F
G
H
Undivided
Division
Mtb/CD56+CD27- cells
Mtb/CD56+CD27+ cells
Mtb/CD56+CD27- cells
Mtb/CD56+CD27+ cells
LTBI-
LTBI+
I
CD69 (%)
CD69 MFI
Unstimulated
Mtb
Mtb+isotipe
Mtb+αCD226
J
CD16 (%)
CD16 MFI
K
HLA-DR (%)
HLA-DR MFI

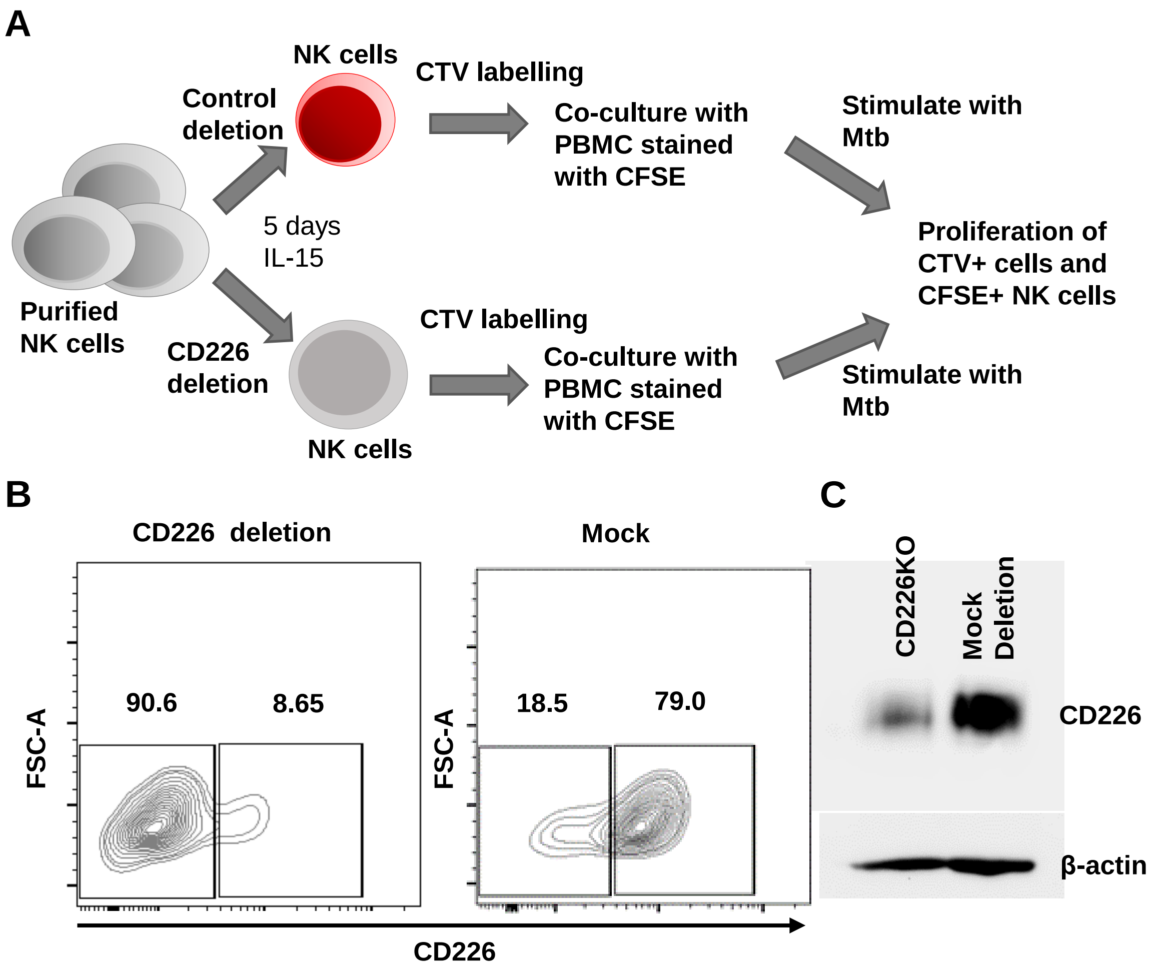
A
NK cells
Control deletion
CTV labelling
Co-culture with PBMC stained with CFSE
Stimulate with Mtb
5 days IL-15
Purified NK cells
Proliferation of CTV+ cells and CFSE+ NK cells
CD226 deletion
CTV labelling
Co-culture with PBMC stained with CFSE
Stimulate with Mtb
NK cells
B
CD226 deletion
Mock
FSC-A
90.6
8.65
18.5
79.0
CD226
C
CD226KO
Mock Deletion
CD226
β-actin

Figure S3

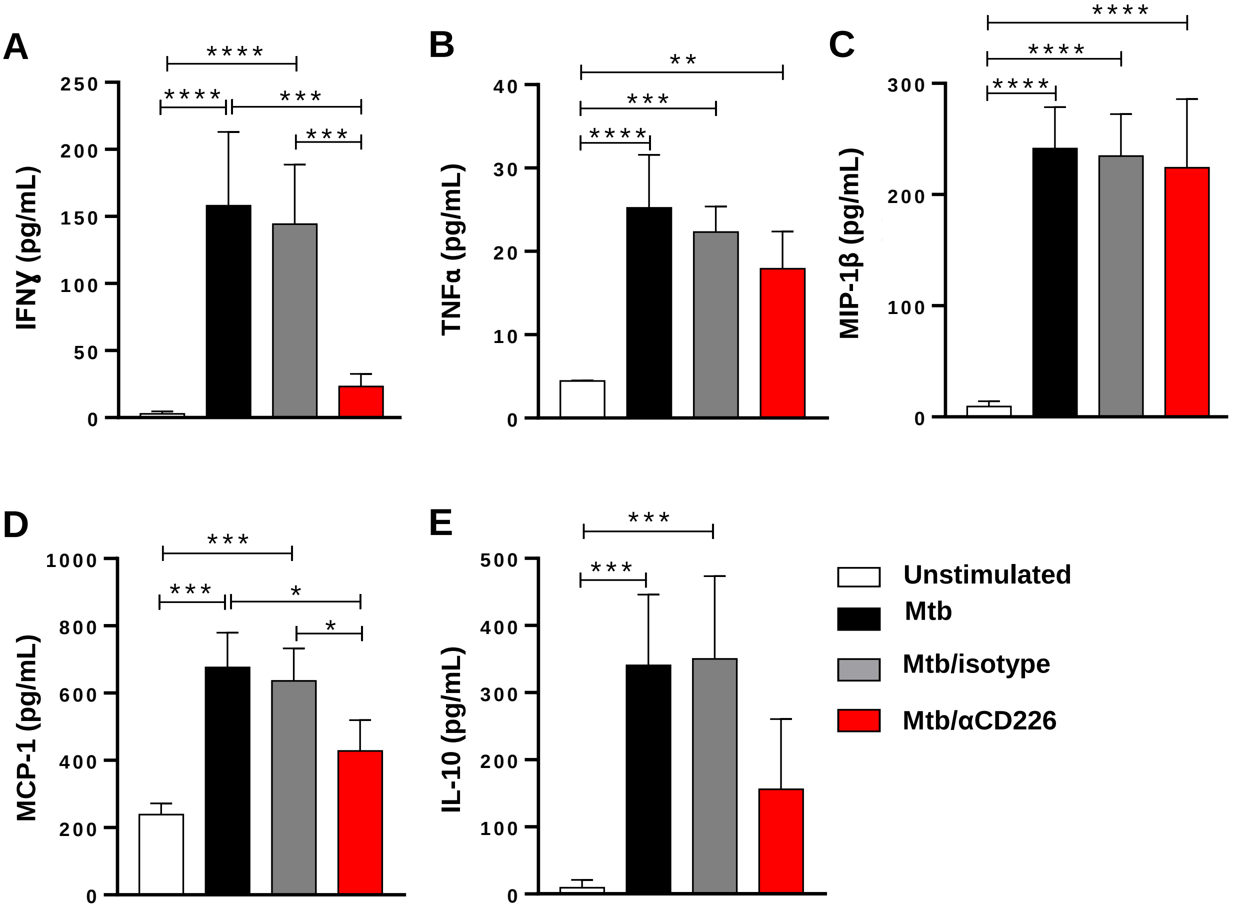
A
B
C
D
E
IFNɣ (pg/mL)
TNFα (pg/mL)
MIP-1β (pg/mL)
MCP-1 (pg/mL)
IL-10 (pg/mL)
Unstimulated
Mtb
Mtb/isotype
Mtb/αCD226

Figure S4

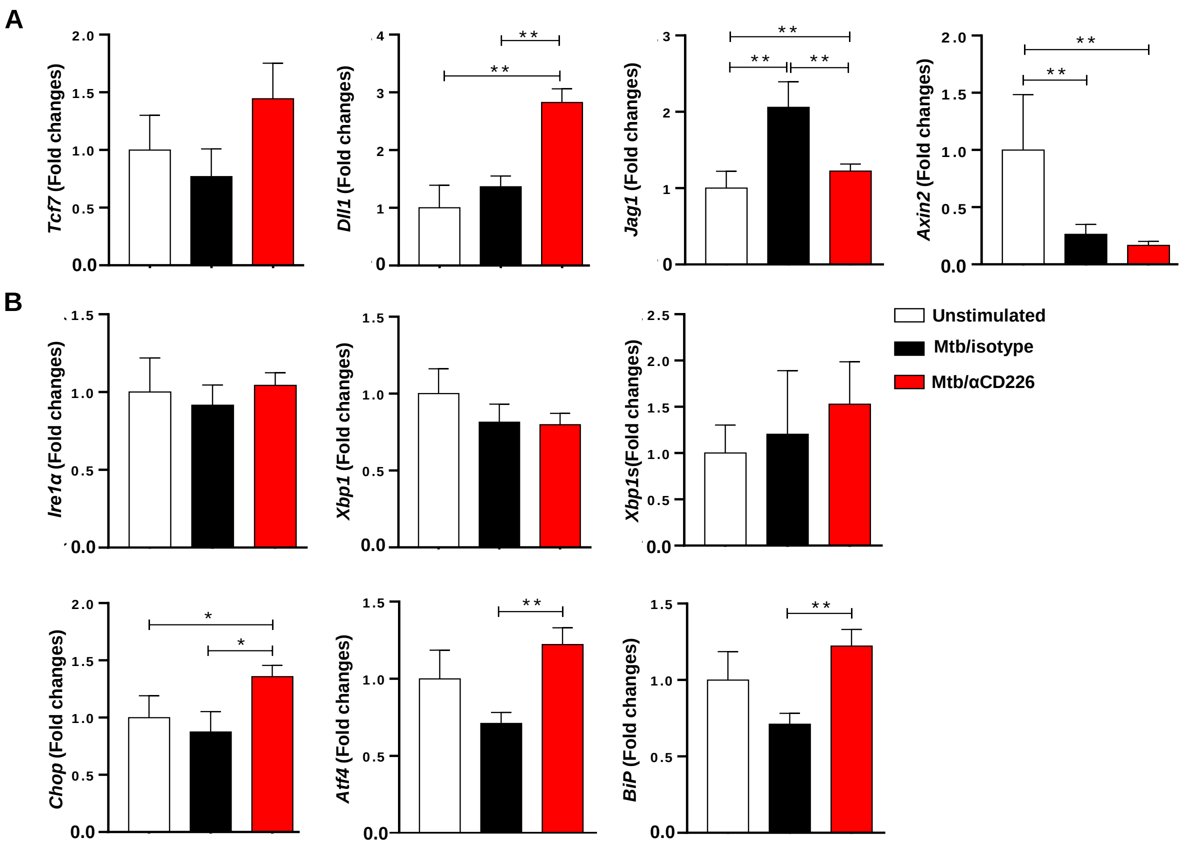
A
B
Tcf7 (Fold changes)
Dll1 (Fold changes)
Jag1 (Fold changes)
Axin2 (Fold changes)
Ire1α (Fold changes)
Xbp1 (Fold changes)
Xbp1s(Fold changes)
Chop (Fold changes)
Atf4 (Fold changes)
BiP (Fold changes)
Unstimulated
Mtb/isotype
Mtb/αCD226

Figure S5

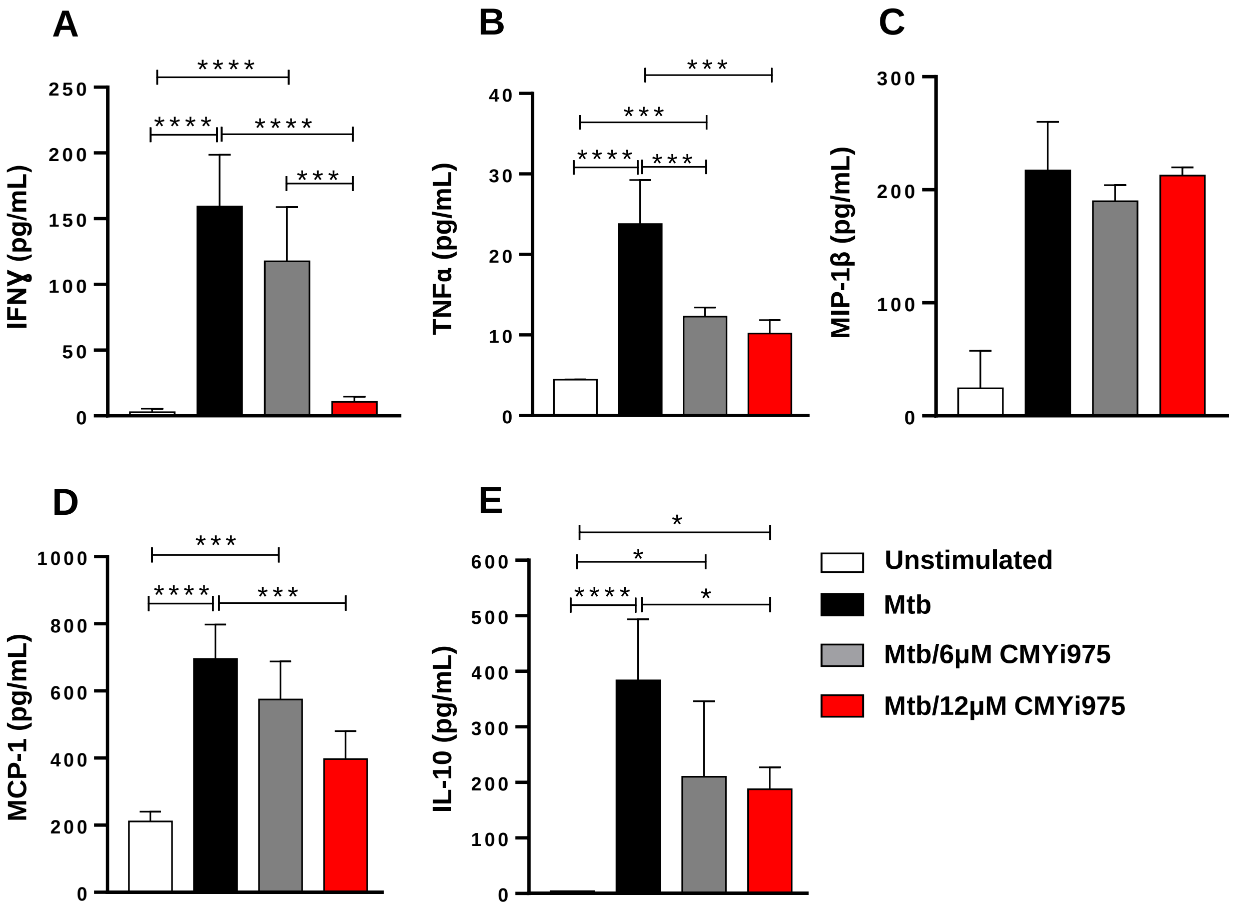

A
IFNɣ (pg/mL)
250
200
150
100
50
0
****
****
****
***
B
TNFα (pg/mL)
40
30
20
10
0
***
***
****
***
C
MIP-1β (pg/mL)
300
200
100
0
D
MCP-1 (pg/mL)
1000
800
600
400
200
0
***
****
***
E
IL-10 (pg/mL)
600
500
400
300
200
100
0
*
*
****
*
Unstimulated
Mtb
Mtb/6μM CMYi975
Mtb/12μM CMYi975

Figure S6

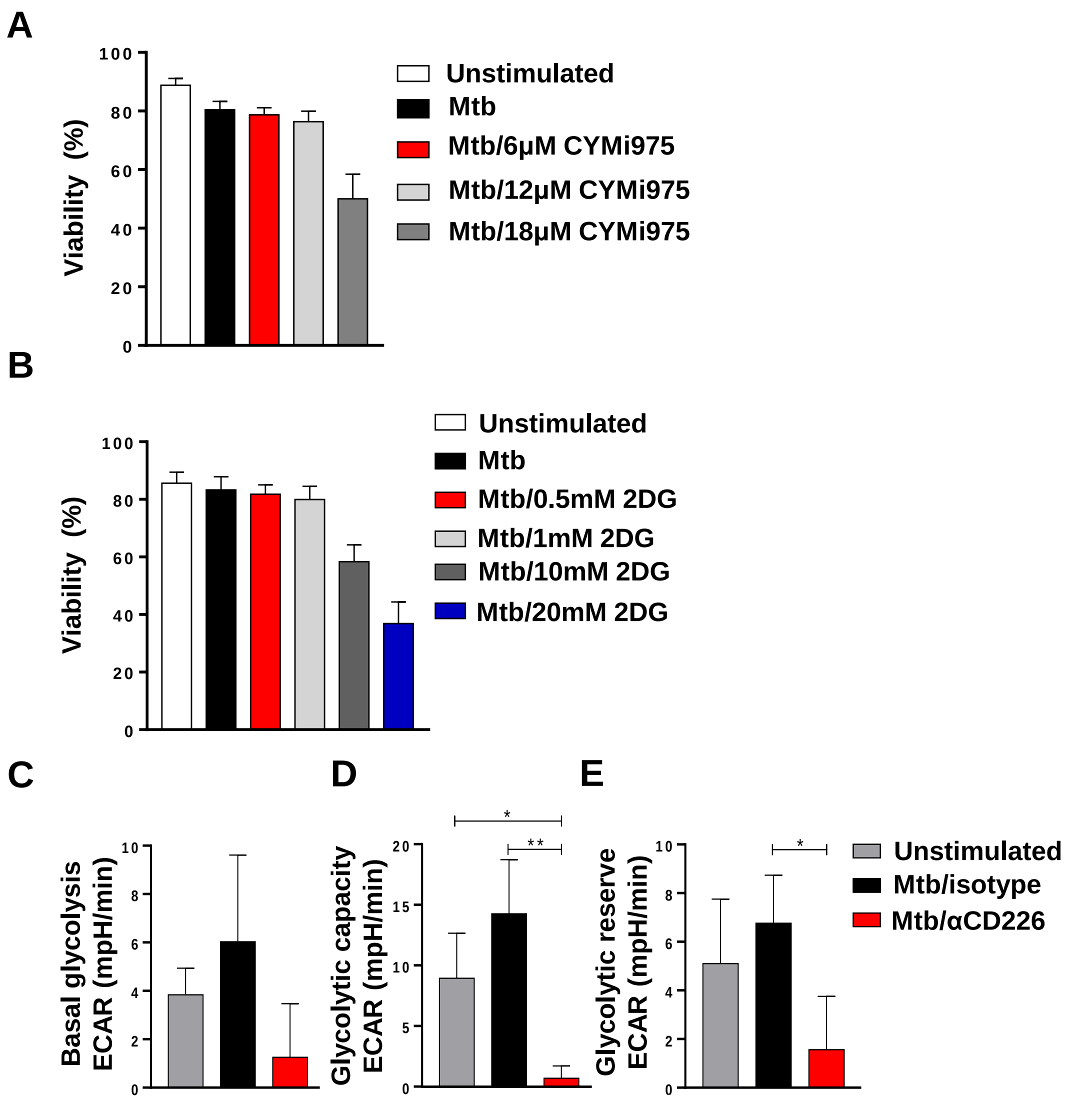
A
Viability (%)
100
80
60
40
20
0
Unstimulated
Mtb
Mtb/6μM CYMi975
Mtb/12μM CYMi975
Mtb/18μM CYMi975
B
Viability (%)
100
80
60
40
20
0
Unstimulated
Mtb
Mtb/0.5mM 2DG
Mtb/1mM 2DG
Mtb/10mM 2DG
Mtb/20mM 2DG
C
Basal glycolysis
ECAR (mpH/min)
10
8
6
4
2
0
D
Glycolytic capacity
ECAR (mpH/min)
20
15
10
5
0
*
**
E
Glycolytic reserve
ECAR (mpH/min)
10
8
6
4
2
0
*
Unstimulated
Mtb/isotype
Mtb/αCD226
